# ^13^C Tracers for Glucose Degrading Pathway Discrimination in *Gluconobacter oxydans* 621H

**DOI:** 10.3390/metabo5030455

**Published:** 2015-09-02

**Authors:** Steffen Ostermann, Janine Richhardt, Stephanie Bringer, Michael Bott, Wolfgang Wiechert, Marco Oldiges

**Affiliations:** Institute of Bio- and Geosciences—IBG-1: Biotechnology, Leo-Brandt-Straße, 52428 Jülich, Germany; E-Mails: s.ostermann@fz-juelich.de (S.O.); j.richhardt@fz-juelich.de (J.R.); st.bringer-meyer@fz-juelich.de (S.B.); m.bott@fz-juelich.de (M.B.); w.wiechert@fz-juelich.de (W.W.)

**Keywords:** microtiter cultivation, ^13^C tracer, pentose phosphate pathway, Entner–Doudoroff pathway, glucose

## Abstract

*Gluconobacter oxydans* 621H is used as an industrial production organism due to its exceptional ability to incompletely oxidize a great variety of carbohydrates in the periplasm. With glucose as the carbon source, up to 90% of the initial concentration is oxidized periplasmatically to gluconate and ketogluconates. Growth on glucose is biphasic and intracellular sugar catabolism proceeds via the Entner–Doudoroff pathway (EDP) and the pentose phosphate pathway (PPP). Here we studied the *in vivo* contributions of the two pathways to glucose catabolism on a microtiter scale. In our approach we applied specifically ^13^C labeled glucose, whereby a labeling pattern in alanine was generated intracellularly. This method revealed a dynamic growth phase-dependent pathway activity with increased activity of EDP in the first and PPP in the second growth phase, respectively. Evidence for a growth phase-independent decarboxylation-carboxylation cycle around the pyruvate node was obtained from ^13^C fragmentation patterns of alanine. For the first time, down-scaled microtiter plate cultivation together with ^13^C-labeled substrate was applied for *G. oxydans* to elucidate pathway operation, exhibiting reasonable labeling costs and allowing for sufficient replicate experiments.

## 1. Introduction

The Gram-negative, strictly aerobic, rod-shaped acetic acid bacterium *Gluconobacter oxydans* has been used for stereo- and regioselective redox reactions in industrial processes for a long time [1,2,3]. Examples of the product spectrum are production of 6-amino-l-sorbose, a key intermediate for the synthesis of the diabetic drug miglitol, the tanning agent dihydroxyacetone from glycerol, and L-sorbose from sorbitol, the first step in the synthesis of vitamin C [4]. Despite its history as a production organism, there still remain a number of open questions about the growth and metabolism of this organism.

Due to the absence of genes encoding for phosphofructokinase and phosphoenolpyruvate synthetase, the Embden–Meyerhoff–Parnas pathway (EMP) and gluconeogenesis are only partially functional [2]. Furthermore, the TCA cycle is truncated due to the absence of succinyl CoA synthetase and succinate dehydrogenase [2].

Genome sequence analysis revealed that *G. oxydans* has the complete gene composition to synthesize all proteinogenic amino acids [2]. Nevertheless, *G. oxydans* grows poorly on a minimal medium lacking proteinogenic amino acids [5,6,7,8]. This is in agreement with the fact that each amino acid itself can be omitted from the cultivation medium, but growth decreases stepwise with an increasing number of missing amino acids (own observation, unpublished). Hence, cultivation with a minimal medium containing L-glutamine as the sole amino acid and glucose as the carbon source led to a significantly reduced growth rate and final OD_600_ [8]. The reason for this behavior is not yet clear, but one can speculate that either the metabolic regulation of *de novo* amino acid biosynthesis is not efficient enough to compensate for missing amino acids or the organism might have a general limitation of energy or carbon precursors in the central metabolism.

*G. oxydans* possesses two spatially separated ways for sugar metabolism. One is located in the periplasm and consists of membrane-bound dehydrogenases, which directly transfer electrons to the respiratory chain via ubiquinone (UQ_10_), and the other in the cytoplasm. The major fraction of carbon and energy source is first oxidized in the periplasm with further metabolization in the cytoplasm, either via the pentose phosphate pathway (PPP) or via the Entner–Doudoroff pathway (EDP). It was demonstrated that the PPP is of major importance for growth on mannitol and glucose [9,10]. Sugar degradation by use of the PPP is energetically more beneficial compared to degradation by EDP [10]. *Gluconobacter oxydans* shows a biphasic growth behavior on glucose [10]. In the first phase glucose is converted to gluconate by periplasmatic oxidation. In the second phase glucose is absent and growth depends on gluconate. The PPP is upregulated in the second growth phase, which was recently confirmed by transcriptomics and ^13^C metabolic flux analysis [11]. Experimental approaches with isotopically labeled tracers like ^13^C-labeled carbon sources are a powerful tool for elucidating the metabolic situation *in vivo*. However, the need for co-metabolization of substrate components like amino acids is barely understood in *G. oxydans* and remains a challenge for such approaches [12,13,14].

In this work we have used PPP and EDP deletion mutants [9] *versus* a *G. oxydans* wild-type strain to study the individual contribution of either pathway to carbon flux within the central metabolism of *G. oxydans*. Both pathways use a unique carbon transition pattern and finally end up in pyruvate, the precursor of e.g., alanine. Hence, alanine mirrors pyruvate ^13^C labeling information and can be easily extracted and measured by LC-MS/MS [15]. By determination of the positional labeling pattern in alanine, PPP or EDP activity can be discriminated. Finally, the growth phase-dependent contribution of each pathway to the metabolism of the reference strain can be derived from these measurements.

For the first time, a ^13^C-labeled substrate together with down-scaled microtiter plate cultivation was applied for *G. oxydans* to elucidate pathway operation, exhibiting reasonable labeling costs and allowing for sufficient replicate experiments. It was shown that with glucose the major degradation occurs via EDP, while the PPP becomes strongly upregulated after glucose depletion when gluconate is used as the substrate. The activation of the PPP is correlated with the secretion of acetate. Due to the lack of phosphofructokinase, fructose 6-phosphate formed in the PPP is isomerized to glucose 6-phosphate and enters the oxidative PPP again. Consequently, a partially cyclic flow of carbon through the PPP occurs and 1 mol glucose 6-phosphate is converted to 1 mol pyruvate and three mol carbon dioxide [11]. This may be a result of cellular energy demand. Furthermore, positional ^13^C labeling of alanine indicates an unusual carbon transition, fueling the hypothesis that there is an unexpected cyclic reaction sequence around the pyruvate node in *G. oxydans*.

## 2. Results

### 2.1. Cultivation of *G. oxydans* at a Small Scale

The present study was conducted to clarify the participation of the two functional pathways for intracellular glucose metabolism, the EDP and the PPP. To this end, *G. oxydans* 621H Δ*upp* as the parental reference strain containing both pathways and three mutant strains was examined [9]. One strain was lacking the genes *edd* (6-phosphogluconate dehydratase, GOX0430) and *eda* (2-keto-3-deoxy-6-phosphogluconate aldolase, GOX0431), thus disabling the EDP. The other two mutant strains were lacking the *gnd* gene (6-phosphogluconate dehydrogenase, GOX1705), thus disabling the PPP, and one of these had in addition an inactive *zwf* gene (glucose 6-phosphate dehydrogenase, GOX0145), but no significant difference in the phenotypes of these two strains was observable under the experimental conditions.

**Figure 1:**
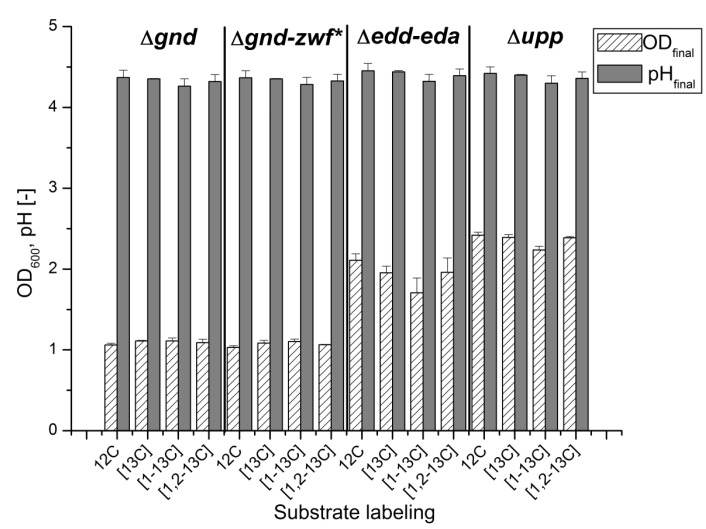
Offline measurement of final OD (white, dashed) and final pH value (grey) after 24 h of cultivation for all combinations of mutant strain and ^13^C-labeled glucose.

All four strains were cultivated in a microtiter plate (MTP) format in the Biolector^®^ on a defined medium with ^13^C-labeled glucose as the carbon and energy source. To exclude an influence of the ^13^C labeling state of the substrate, all four strains were cultivated with differently labeled species of glucose in at least two biological and three technical replicates. The measurement of the growth phenotype (final pH and OD_600_) did not reveal any influence of the substrate labeling state, as shown in Figure 1.

All strains showed a very similar final pH value of 4.5. This is in agreement with the fact that all strains had consumed the glucose completely (data not shown), which led to a drop in the pH due to the formation of gluconic and acetic acid. As already observed in bioreactor cultivations, the strains Δ*upp* Δ*gnd* and Δ*upp* Δgnd-zwf*, both lacking the PPP, attained only half of the final OD_600_ of the strain lacking the EDP and the reference strain, giving a first hint of the importance of PPP in the second growth phase [10].

### 2.2. Transition of ^13^C Labeling from Glucose to Alanine by *G. oxydans* Metabolism

Glucose is either oxidized by the membrane-bound glucose dehydrogenase (GOX0265, *gdhM*) to gluconate or taken up by the cells via an unknown transporter system, as the PTS system is considered to be inactive due to the absence of the required EIIB and EIIC subunits. Gluconate is taken up by the cells via a putative gluconate permease (GOX2188) [2]. Cytoplasmic catabolism of glucose can occur via glucose dehydrogenase (GOX2015, *gdhS*) or glucose kinases (GOX2419, *glkA* and GOX1182, *glkB*) [10].

**Figure 2:**
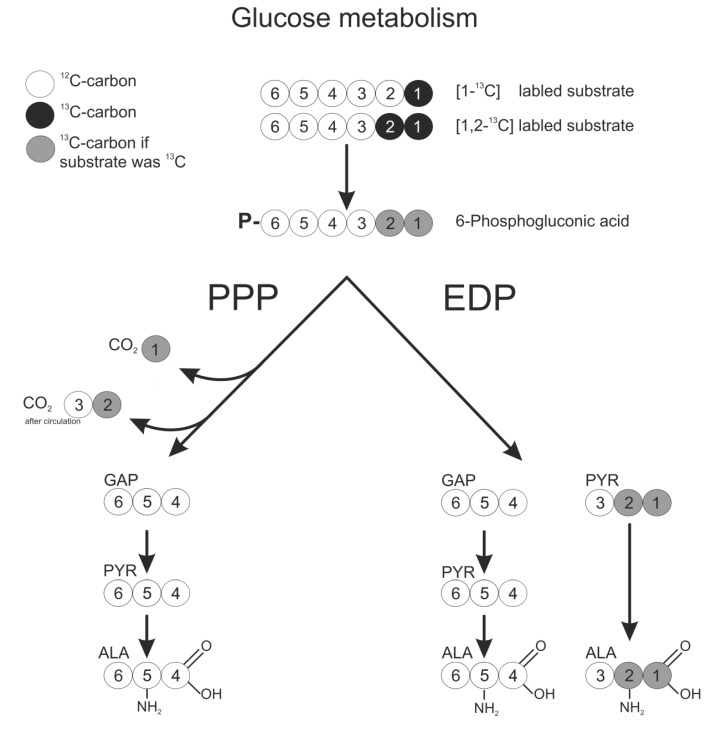
Transition scheme of glucose via the pentose phosphate pathway (PPP) and the Entner–Doudoroff pathway (EDP). The circles indicate carbon atoms in the isotopic states ^12^C (white) and ^13^C (black); depending on the substrate, the products may contain the ^13^C labeling (grey). The numbers of the carbon position in glucose is based on IUPAC nomenclature. The numbers in the remaining metabolites originate from the glucose carbon position.

For the labeling approach used in the present work, it is irrelevant whether single steps of the conversion from glucose to 6-phosphogluconate occur intra- or extracellularly, since the carbon backbone is not altered by this conversion. Intracellular gluconate can be converted to 5-ketogluconate by a gluconate-5-dehydrogenase (*gno*, GOX2187), or phosphorylated by a gluconate kinase (*gnk*, GOX1709) [2,16]. The metabolite 6-phosphogluconate (6-PG) exclusively serves as a precursor molecule for both intracellular glucose degradation pathways, *i.e.*, PPP and EDP, and ends up in the common C_3_-molecule pyruvate. All carbon transition information was deduced with the Omix [17] software tool from the KEGG database [18]. In Figure 2, the schematic carbon transition is displayed.

The six carbon atoms of 6-PG are converted by the EDP into two molecules. One molecule of pyruvate, containing C1–C3 of the 6-PG precursor, and one molecule of glyceraldehyde-3-phosphate (GAP), being composed of C4–C6 of 6-PG, are produced. In case of degradation by the PPP, only the last three carbon atoms C4–C6 of 6-PG are converted to GAP and finally to pyruvate. The C1 carbon of 6-PG is directly decarboxylated by 6-phosphogluconate dehydrogenase (GND) to CO_2_. The remaining two carbon atoms, C2 and C3, are drained into biomass or turned into the C1 position of fructose-6-phosphate, which is again split off by GND in the next oxidative PPP cycle. Both pathways generate one molecule of GAP per glucose, which is finally converted to pyruvate, the primary precursor of alanine [19]. The GAP-pyruvate conversion occurs without change in the carbon backbone of the molecules and therefore without altering the ^13^C labeling information.

Alanine is synthesized by transamination of pyruvate without change in the carbon backbone and thus positional labeling information. The genome encodes two possible enzymes involved in pyruvate transamination, the serine-pyruvate transaminase EC 2.6.1.51 encoded by GOX1298 and another transaminase (EC.2.6.1.2) encoded by GOX0190. The second gene is annotated as aspartate aminotransferase but has 31% amino acid identity to an alanine transaminase in the strain *Escherichia coli* K-12 MG1655 (YP_190636.1) and contains conserved regions of the AAT_I superfamily.

### 2.3. ^13^C labeling Experiments for Pathway Discrimination

The schematic reactions from 6-phosphogluconate to alanine are presented in Figure 2, including the labeling pattern of alanine with respect to the used labeled glucose and the catabolic pathway. Labeling experiments were performed using four differently labeled variants of glucose, summarized in Table 1. The nomenclature of ^13^C labeling states of the metabolites is presented in Section 4.5.

*^12^C control with unlabeled glucose.* All four mutant strains showed the expected natural isotope abundance with comparable values for m.0 and m.1 respectively. This ratio was used for adjusting all labeling measurements for the alanine pattern of the preculture cells in the applied washout correction.

**Table 1:** Mass isotopomer fractions in alanine of the four strains Δ*gnd*, Δ*gnd* zwf*, Δ*edd*-*eda*, and Δ*upp* cultivated with differently ^13^C labeled substrates. The isotopic state of alanine can be unlabeled (m.0), single (m.1), double (m.2), or triple (m.3) ^13^C labeled. All values were generated from at least two biological replicates with two technical replicates each. The values for (^13^C) samples represent six biological replicates each. All values are corrected for their inoculation labeling pattern. The nomenclature of the ^13^C labeling states of the metabolites is presented in Section 4.5.

		Mass Spectrometry Signal Resp. Labeling State of Alanine			
Substrate	Strain	m.0	m.1	m.2	m.3
^12^C control	Δ*gnd*	0.96 ± 0.001	0.04 ± 0.001	n.d.	n.d.
	Δgnd-zwf*	0.96 ± 0.001	0.04 ± 0.001	n.d.	n.d.
	Δe*dd-eda*	0.96 ± 0.001	0.04 ± 0.001	n.d.	n.d.
	Δ*upp*	0.96 ± 0.001	0.04 ± 0.001	n.d.	n.d.
[^13^C] control	Δ*gnd*	0.37 ± 0.014	0.02 ± 0.001	0.07 ± 0.002	0.54 ± 0.015
	Δgnd-zwf*	0.37 ± 0.009	0.02 ± 0.001	0.08 ± 0.004	0.54 ± 0.008
	Δe*dd-eda*	0.30 ± 0.013	0.03 ± 0.002	0.08 ± 0.003	0.59 ± 0.01
	Δ*upp*	0.31 ± 0.01	0.02 ± 0.008	0.08 ± 0.005	0.59 ± 0.009
[1-^13^C]	Δ*gnd*	0.51 ± 0.011	0.47 ± 0.011	0.01 ± 0.000	n.d.
	Δgnd-zwf*	0.50 ± 0.014	0.49 ± 0.014	0.02 ± 0.000	n.d.
	Δe*dd-eda*	0.96 ± 0.000	0.04 ± 0.000	n.d.	n.d.
	Δ*upp*	0.76 ± 0.004	0.24 ± 0.003	0.01 ± 0.000	n.d.
[1,2-^13^C]	Δ*gnd*	0.45 ± 0.016	0.09 ± 0.004	0.45 ± 0.013	0.01 ± 0.000
	Δgnd-zwf*	0.44 ± 0.031	0.09 ± 0.006	0.46 ± 0.024	0.01 ± 0.001
	Δe*dd-eda*	0.95 ± 0.001	0.05 ± 0.001	n.d.	n.d.
	Δ*upp*	0.64 ± 0.006	0.13 ± 0.002	0.22 ± 0.004	0.01 ± 0.001

*[^13^C] control with fully labeled glucose*. If there were no other pyruvate fueling reactions besides glucose degradation, the labeling state of alanine could be expected to be fully ^13^C labeled (m.3) in all mutant strains when fed with uniformly labeled [^13^C] glucose. However, an influx of 37% unlabeled alanine for the strains Δ*gnd* and Δgnd–zwf* and 30% for the strains Δ*edd-eda* and parent strain, respectively, was measured. This indicates that besides ^13^C glucose, unlabeled amino acids from the culture medium are also taken up and degraded to pyruvate, illustrating the challenge of cosubstrate metabolization. Since alanine was lacking in the culture medium, the fueling reactions for unlabeled pyruvate must depend on other amino acids, e.g., serine, which is converted to pyruvate by serine deaminase (GOX0794).

The four strains can be grouped into absent PPP (Δ*gnd* and Δgnd–zwf*) with 37% m.0 values, and active PPP (Δ*edd-eda* and parent strain) with 7% lower m.0 values, respectively. Both groups show remarkably similar values within the error range. An absent PPP seemed to result in a higher demand for amino acid degradation, although the final OD was only 50% of the strains with active PPP (Figure 1). Reasons could be the lower energy yield of glucose degradation by the EDP instead of the PPP or the gluconeogenetic formation of PPP intermediates for biomass formation and thus an increased amino acid catabolism.

*[1-^13^C]-labeled glucose.* This substrate should result in 50/50 labeling distribution of unlabeled and single labeled alanine for the strains lacking PPP, and completely unlabeled alanine for the strain lacking EDP. For the PPP negative strains Δ*gnd* and Δgnd-zwf*, the ^13^C-labeled C1 carbon from glucose is transferred into the C1 position of the first pyruvate. Due to the unlabeled second pyruvate generated from GAP, the observed results are close to the labeling ratio of 50/ 50 with a little more m.0 (m.0 = 50.5, m.1 = 48). A more detailed picture for the single ^13^C labeling is given in Table 2. The m.1.1 lane indicates alanine molecules with a single ^13^C labeling at the carbon position C2 or C3. With 2%, this value is close to the natural labeling abundance. Consequently the m.1.0 isotopomer shows that 46% of all measured alanine molecules are specifically labeled at carbon position C1, as predicted by the labeling transition model. For the Δ*edd-eda* mutant strain the ^13^C-labeled C1 carbon from glucose should be split off through decarboxylation by entering the PPP, resulting in alanine isotope enrichments comparable to the ^12^C control, which was achieved. The results for the Δ*upp* strain (m.0 = 0.76, m.1 = 0.24) are very interesting, since they are a superposition of both pathway phenotypes, clearly indicating that PPP and EDP are both involved in glucose degradation in the experiments.

**Table 2:** Fragmentation pattern of alanine for (1-^13^C)- and (1,2-^13^C)-labeled glucose. The nomenclature of the ^13^C labeling states of the metabolites is presented in Section 4.5.

		Mass Spectrometry Signal Resp. Labeling State of Alanine					
Substrate	Strain	m.0	m.1.0	m.1.1	m.2.1	m.2.2	m.3.2
[1-^13^C]	Δ*gnd*	0.51 ± 0.011	0.45 ± 0.011	0.02 ± 0.000	0.01 ± 0.000	n.d.	n.d.
	Δgnd-zwf*	0.50 ± 0.014	0.47 ± 0.014	0.02 ± 0.000	0.02 ± 0.000	n.d.	n.d.
	Δ*edd-eda*	0.96 ± 0.000	0.01 ± 0.000	0.03 ± 0.000	n.d.	n.d.	n.d.
	Δ*upp*	0.76 ± 0.004	0.21 ± 0.004	0.03 ± 0.000	0.01 ± 0.000	n.d.	n.d.
[1,2-^13^C]	Δgnd	0.45 ± 0.016	0.00 ± 0.002	0.08 ± 0.005	0.45 ± 0.013	n.d.	0.01 ± 0.000
	Δgnd-zwf*	0.44 ± 0.031	n.d.	0.08 ± 0.006	0.46 ± 0.024	n.d.	0.01 ± 0.001
	Δ*edd-eda*	0.95 ± 0.001	0.01 ± 0.000	0.04 ± 0.001	n.d.	n.d.	n.d.
	Δ*upp*	0.64 ± 0.006	0.05 ± 0.001	0.08 ± 0.001	0.21 ± 0.004	0.01 ± 0.000	0.01 ± 0.001

*[1,2-^13^C] labeled glucose.* This substrate was expected to result in labeling patterns comparable to those for ^13^C_1_-labeled glucose due to the similarity in the carbon transition. The PPP negative strains Δ*gnd* and Δgnd-zwf* showed with 45% m.0 and 45% m.2 the same ratio of unlabeled and double labeled alanine molecules as observed for ^13^C_1_-labeled glucose. However, 9% of single-labeled alanine was an unexpected finding. The more detailed fragmentation pattern (Table 2) indicated that nearly the whole signal stemmed from m.1.1. Hence, the ^13^C-labeled carbon atom is located at carbon position C2 or C3. An explanation could be a decarboxylation of the ^13^C-labeled C1 carbon atom of alanine or a precursor and a subsequent bonding of a naturally labeled carbon atom at this position. Such a potential reaction cycle would be concealed by the substrate [1-^13^C] glucose, since a ^12^C carbon exchange at position C1 would result in unlabeled alanine. The Δ*edd-eda* strain showed, with 95% m.0 and 5% m.1, respectively, high similarity to the expected natural labeling abundance. The results of the Δ*upp* strain again represent a mixture of both pathway phenotypes concerning the m.0 and m.2 lane. This clearly points out that both pathways, PPP and EDP, were active in glucose catabolism. However, 13% signal for the m.1 lane was again an unexpected finding and a strong hint that an essential reaction concerning the pyruvate node in *G. oxydans* still remains to be elucidated.

The described results show that the mutant strains and substrate combinations used are suitable to prove participation of both pathways, PPP and EDP, in intracellular glucose degradation in the Δ*upp* reference strain. Nevertheless, the control with uniformly labeled [^13^C] glucose indicated that besides glucose degradation additional pyruvate fueling reactions occur. In addition, the specific drain of GAP into biomass formation cannot be quantified with the setup used and thus it is difficult to determine a reliable pathway split ratio of the metabolic flux among PPP and EDP.

### 2.4. Time Resolved ^13^C Labeling Experiments

The results from labeling experiments clearly illustrated that PPP and EDP are both active during cultivation with glucose. Due to the biphasic growth behavior of *G. oxydans*, the question arose of whether PPP and EDP might have growth phase-specific functions. To gain insight into whether there was a growth phase-dependent pathway activity, the PPP negative strain Δ*gnd* and the parent strain Δ*upp* were cultivated with [1,2-^13^C] glucose for 24 h and sampled every 2 h.

**Figure 3:**
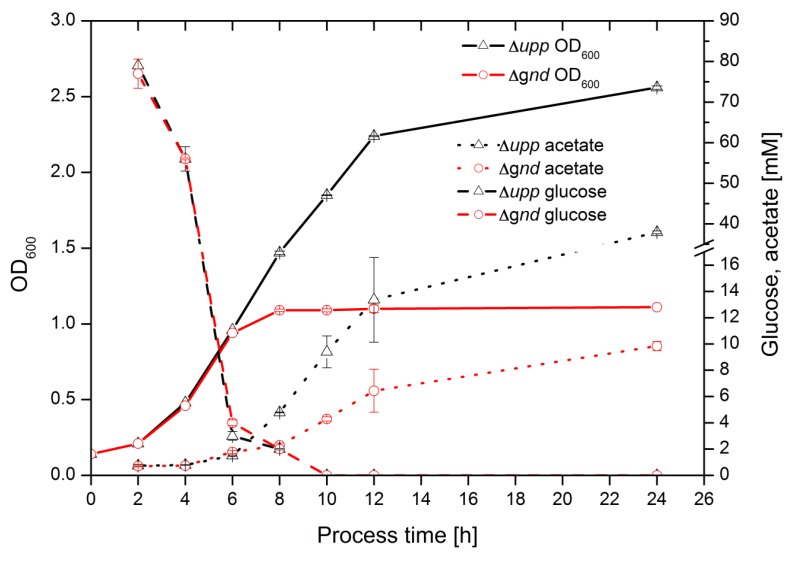
Growth (full lines) of Δ*upp* (Δ, black) and Δ*gnd* (o, red) strains. Glucose (dashed lines) and acetate (dotted lines) concentration in supernatant at a sequentially sampled cultivation.

The offline measured values for OD, glucose consumption, and acetate secretion are displayed in Figure 3. In the first 6 h, glucose consumption and acetate formation were identical for both strains and likewise the growth rate. After 6 h of cultivation, the glucose consumption was dramatically reduced, matching a reported threshold of 6–8 mM glucose concentration to induce a glucose transport system in other organisms [20,21]. It took another 4 h to completely consume the glucose present in the culture medium.

After 6 h, the Δ*gnd* strain showed a drastic reduction of its growth rate and reached its final OD of 1.1 ± 0.01 two hours later, whereas the parent strain exhibited only a slightly reduced growth rate after 6 h and grew to an OD of 2.56 ± 0.01. Acetate excretion started after 4 h and the parent strain produced nearly a four-fold higher concentration after 24 h compared to the Δ*gnd* strain. Concerning these data, it can be assumed that with glucose falling below a certain concentration the metabolic activity was changing. The PPP-negative mutant Δ*gnd* was no longer able to grow, so it can be concluded that PPP plays an essential role in the second growth phase.

**Figure 4:**
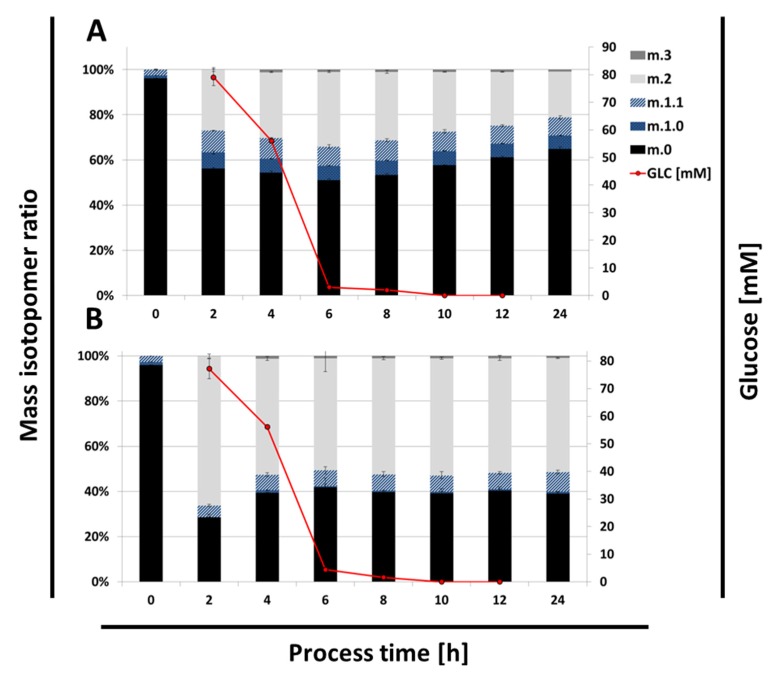
Mass isotopomer proportions of the ^13^C labeling states of alanine for (**A**) Δ*upp* and (**B**) Δ*gnd* mutant strains. The process time indicates the sampling points; the red line indicates the respective glucose concentration. The isotopic state of alanine can be unlabeled (m.0, black), single (m.1.0 blue dotted, m.1.1 blue dashed), double (m.2, grey), or triple (m.3, dark grey) ^13^C labeled. Mean values and standard deviations of at least two replicates are shown.

The specific labeling distribution in alanine, formed after degradation of [1,2-^13^C] glucose, is shown in Figure 4. For the PPP-negative Δ*gnd* strain, with EDP as the exclusive catabolic pathway, a 50/50 mixture of m.0 and m.2 signal was expected due to the formation of unlabeled GAP, double-labeled pyruvate, and, subsequently, alanine. Two hours after inoculation, 66% of alanine m.2 was found (Figure 4B). After an additional two hours, this was reduced to the expected theoretical value of 51%, then staying constant during the remaining cultivation. The signal for m.0 is a mixed signal from GAP and degraded unlabeled amino acids from the cultivation medium. It cannot be determined from the data whether the conversion rate of GAP to pyruvate or the uptake rate of precursors from the cultivation medium was changing. The observed reduction of growth rate after 6 h cannot be detected by the labeling state of alanine. Hence, a potential change of intracellular metabolism did not affect the alanine labeling state, *i.e.*, the fueling and composition of the pyruvate pool. Identical growth rates for the PPP-negative strain Δ*gnd* and the strain Δ*upp* in the first 6 h show without a doubt that EDP pathway activity is sufficient for *G. oxydans* and PPP is not essential for growth on glucose in the first phase, as was also shown in bioreactor cultivations with higher substrate concentrations [10].

For the Δ*upp* strain, the theoretical labeling values for either exclusive PPP or EDP pathway activity would be 0% and 50% (as observed for the Δ*gnd* strain), respectively. As shown in Figure 4A, the labeling pattern can be separated into two phases. In the first 6 h, with glucose available the amount of m.2 labeled alanine increased by up to 33%. This can be directly correlated with increasing EDP activity. Although Olijve and Kok [8] described a complete repression of PPP under glucose-rich conditions, the labeling data clearly show a contribution of PPP to the glucose catabolism, otherwise higher m.2 mass isotopomer would have been present. Thus, it can be concluded that EDP and PPP pathways are both involved in glucose degradation in the first growth phase, confirming model-based results [11].

After 6 h, when glucose availability became limiting, the amount of m.2 labeling continuously decreased, down to 20% after 24 h (Figure 4A). This could be explained by an increasing degradation of unlabeled amino acids from the medium or a shift of the PPP/EDP split ratio towards PPP. The shift to PPP is coherent with the findings of Hanke *et al.* [11], showing a strong upregulation of PPP in the second growth phase on gluconate. Nevertheless, the data still indicate a relevant EDP contribution (otherwise m.2 would be diminished in that phase), but the PPP clearly is more important in this second growth phase on gluconate. This ultimate relevance of the PPP for the second growth phase is undoubtedly proven, since the PPP-negative Δ*gnd* strains completely lacked a second growth phase and were not able to utilize gluconate for further biomass formation.

For both strains (Δ*upp* and Δ*gnd*) a surprising signal for single-labeled alanine was detectable after degradation of [1,2-^13^C] glucose, which was not expected due to the network topology. In the Δ*gnd* strain, single-labeled alanine is observed exclusively as an m.1.1 mass isotopomer, indicating ^13^C labeling at carbon position C2 or C3. Since the relative amount of 7 ± 1% single-labeled alanine did not change over the whole cultivation time, it seems to be a growth phase-independent reaction. As mentioned before, a specific C1 decarboxylation of double-labeled pyruvate with subsequent attachment of an unlabeled carbon monomer would be a possible explanation for these signals.

The Δ*upp* parent strain showed with 8.7 ± 0.7% a slightly increased amount of m.1.1. Furthermore, the mass isotopomer m.1.0 with 6.3 ± 0.4% was detectable in contrast to the Δ*gnd* strain, indicating a specific ^13^C labeling at alanine carbon position C1. With regard to the network structure, this m.1.0 isotopomer seems to involve a carboxylating reaction, *i.e.*, ^13^CO_2_ could be ligated to an unlabeled C2 compound. The ratio between m.1.0 and m.1.1 over the cultivation time in the Δ*upp* strain is predominantly constant, again indicating that this unknown metabolic process is not growth phase-dependent and might be at least partially PPP associated (m.1.0).

## 3. Discussion

In this work we addressed the question of whether labeling experiments with *G. oxydans* can be performed at a small scale to elucidate the participation of the two functional pathways for glucose metabolism, the EDP and the PPP. Apart from gaining knowledge on intracellular flux distribution, the aim of this work was to demonstrate the applicability of the novel microscale method for pathway analysis in *G. oxydans*.

The labeling patterns shown indicate that the used network topology, especially for the PPP, EDP, and incomplete EMP, was correct, as nicely shown by the match between experimental labeling *versus* theoretical prediction for the PPP and EDP in the reference experiments. The loss of labeling in the EDP-negative Δ*edd-eda* strain when fed with [1-^13^C] or [1,2-^13^C] glucose matched 100% PPP pathway activity and was in agreement with the lack of 6-phosphofructokinase [2].

A control experiment with uniformly [13C]-labeled glucose showed a metabolic influx of naturally labeled substrates up to 37% into alanine for the strains lacking the PPP and 30% for the Δ*upp* parent strain and the Δ*edd-eda* mutant, demonstrating that supplemented amino acids from the medium are co-metabolized besides glucose. This illustrates the challenge of applying methods using isotopically labeled substrate if a suitable minimal culture medium is not available, especially for retrobiosynthetic approaches like this one. The amino acid uptake supports the observation of increasing maximal growth rate μ_max_ and biomass yield dependent on the number of supplemented amino acids (unpublished data), pointing to the question of whether the provision of additional energy or the provision of additional carbon sources is responsible for the positive influence of amino acid supplementation on growth.

In the presence of glucose, the periplasmatic oxidation from glucose to gluconate directly fuels the respiratory chain with electrons, allowing ATP formation by oxidative phosphorylation. The energy content of gluconate is less than that of glucose, since periplasmatic oxidation already cleaved hydrogen from the substrate. Hence, the energy and redox state of the gluconate substrate might not be sufficient to serve *G. oxydans* metabolism in the PPP negative Δ*gnd* strain. An interesting difference between the usage of the PPP and the EDP is energetic efficiency. Under the assumption of complete degradation of glucose to acetate without side reactions, the conversion of 1 mol 6-phosphogluconate via the EDP yields 2 mol acetate + 2 mol CO_2_ + 3 mol NAD(P)H + 2 mol ATP, while a cycling PPP degrades 1 mol 6-phosphogluconate to 1 mol acetate + 4 mol CO_2_ + 6 mol NAD(P)H + 2 mol ATP [22]. An upregulation of PPP in the second growth phase would allow regeneration of 60% more reduced cofactors compared to the EDP. Hence, PPP is energetically more effective for metabolism, at the expense of higher loss of carbon dioxide. Energy supply seems to be a major regulatory motive of *G. oxydans* and potential insufficient energy supply of the PPP negative Δ*gnd* mutant in the second growth phase on gluconate might be the reason for the absence of further growth. Our data comply with the results of a characterization of enzymes involved in the central metabolism of *Gluconobacter oxydans*, pointing to a major role of the pentose phosphate pathway in the intermediary metabolism of *G. oxydans*, while the Entner–Doudoroff pathway was found to contribute only to a minor extent during growth on gluconate in the second phase [23].

The periplasmatic conversion speed of glucose to gluconate is strongly reduced when glucose reaches a concentration of 5 mM, as reported by Olijve and Kok [8]. At the same time the growth rate is reduced and acetate secretion starts. Under these conditions the cells probably redirect the carbon flow from pyruvate to acetic acid, either due to a reduced precursor demand for biomass synthesis or to an increased cellular energy demand [24]. The oxidation of acetaldehyde, formed by pyruvate decarboxylase [25,26], delivers acetate and one reduced cofactor equivalent NAD(P)H, which could be the driving force for acetate formation in the second growth phase. Since the Δ*gnd* strain still secretes acetate after having stopped biomass formation, the redox equivalents seem to be used for maintenance or other energy-consuming reactions.

The labeling data generated with [1,2-^13^C] glucose revealed an unusual ^13^C labeling pattern in the m.1.0 and m.1.1 mass isotopomers. The stable amounts of m.1-labeled alanine for the continuously sampled strains Δ*upp* and Δ*gnd* show that the responsible reaction is growth phase independent and continuously active at a constant rate. This gives rise to the conclusion about a potential futile cycle around the pyruvate node in *G. oxydans*.

An explanation for the labeling pattern could be 1) decarboxylation of pyruvate carbon position C1 with loss of one ^13^C, and 2) the addition of a naturally labeled single carbon at the remaining C_2_ molecule. Such a reaction would generate single-labeled alanine at carbon position C2 or C3, resulting in the observed m.1.1 alanine signal. In contrast to Δ*gnd* mutant strains*,* the PPP-active Δ*upp* strain additionally generated m.1.0-labeled alanine, indicating a specific C1 attachment of a ^13^C-labeled carbon at a naturally labeled C_2_ molecule. The first PPP-devoted reaction catalyzed by 6-phosphogluconate dehydrogenase specifically cleaves off the C1 atom of 6-PG, and in the case of [1-^13^C]- and [1,2-^13^C]-labeled glucose this results in ^13^CO2 formation, indicating that CO2 could be involved as the C1-unit in this transformation. This potential cycling is masked if ^12^C-, [^13^C]-, or [1-^13^C]-labeled glucose is used as the carbon source. When using [1-^13^C] glucose, the labeling information is located at carbon position C1. If the described reaction sequence occurs, the resulting molecule is masked as an unlabeled m.0 species due to the attachment of an unlabeled CO_2_ at position C1. This would lead to an elevated signal for the m.0 lane, which can indeed be observed for the strains Δ*gnd*, Δgnd-zwf*, *and* Δ*upp* (Table 1 and Table 2)*.* Consequently, for the [1-^13^C]-labeled substrate the ratio of unlabeled (m.0) to labeled (m.1, m.2, m.3) alanine is higher than the [1,2-^13^C]-labeled substrate.

Several reactions, mainly around the pyruvate node, were checked as probable candidates for generating the observed labeling results. The side reaction of 2-keto-3-deoxy-6-phosphogluconate aldolase encoded by *eda* (GOX0430), *i.e.*, decarboxylation of oxaloacetate to pyruvate, can be excluded since the resulting labeling transition does not match these results. The same holds true for the non-oxidative branch of the PPP catalyzed by transketolase and transaldolase, encoded by GOX1703 and GOX1704. The decarboxylation reactions from pyruvate to acetyl-CoA or acetaldehyde generate a suitable reactive C_2_ molecule, but no reaction sequence back to pyruvate could be identified starting from acetyl-CoA.

**Figure 5:**
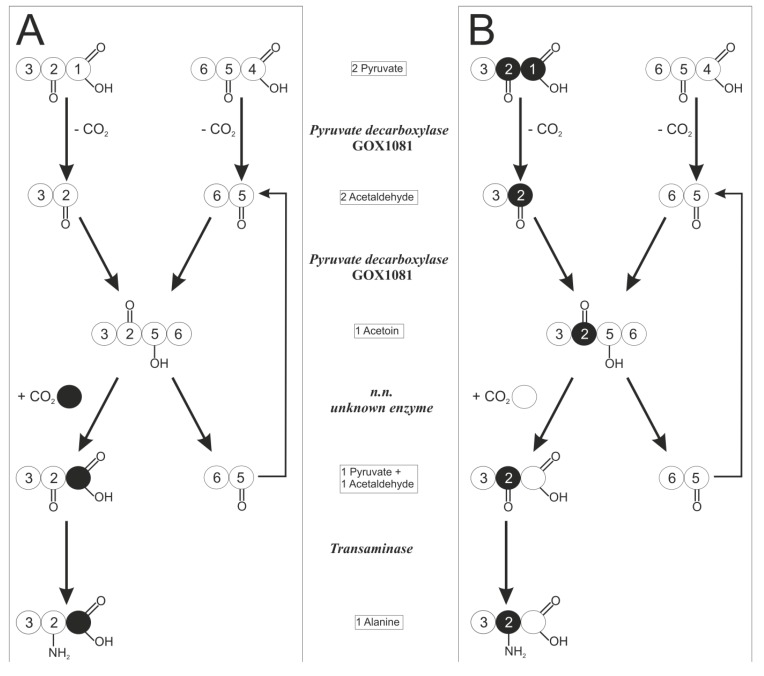
Scheme of a hypothetical reaction sequence from pyruvate via acetoin back to pyruvate, including the carbon transition. Atoms are labeled ^13^C (black) or ^12^C (white). The numbers originate from carbon position in the substrate glucose: (**A**) unlabeled precursors and ^13^C-labeled CO_2_ transformed to alanine generating m.1.0 signal; (**B**) double-labeled pyruvate originated from [1,2-^13^C]-labeled glucose transformed to alanine, generating an m.1.1 signal.

For acetaldehyde, a hypothetical reaction sequence explaining the observed labeling data was identified. The constitutively expressed pyruvate decarboxylase (*pdc*, GOX1081) converts 1 mol pyruvate to 1 mol acetaldehyde and 1 mol CO_2_ as main reaction, but is also able to condense 2 mol acetaldehyde to 1 mol acetoin under suitable reaction conditions [27], as shown in Figure 5. Next, a thus-far unspecified enzyme would have to carboxylate acetoin and simultaneously split the carboxylation product into one pyruvate and one acetaldehyde. Such a reaction sequence would allow the cell to get rid of toxic acetaldehyde and recover CO_2_. The resulting transition of ^13^C labeling would be a perfect match for the observed m.1.0 (Figure 5A) and m.1.1 (Figure 5B) mass isotopomers.

The reverse reaction, *i.e.*, the conversion of pyruvate and acetaldehyde to acetoin and carbon dioxide, has been described as a side activity of acetolactate synthase in *Neurospora crassa* by Kuwana *et al.* [26]. Nevertheless, this enzyme is not able to catalyze the opposite direction, *i.e.*, the carboxylation of acetoin with carbon dioxide leading to pyruvate and acetaldehyde, since thermodynamics undoubtedly favor the decarboxylation. To conclude, the putative enzyme converting acetoin and carbon dioxide to pyruvate and acetaldehyde would require energy to shift the reaction equilibrium to the formation of pyruvate and acetaldehyde.

Peters *et al.* [16] showed that a deletion of *pdc* leads to an export of pyruvate instead of acetate, indicating Pdc activity at least in the second growth phase. Furthermore, they observed an increased final cell density in the Δ*pdc* mutant compared to the Δ*upp* strain. It was speculated that the absence of toxic acetaldehyde was the reason for this observation. It has been reported that *G. oxydans* has the potential to produce large quantities of aldehydes [2,28] and that a detoxification system is present, of which single enzymes are described [29,30,31]. Schweiger *et al.* [30] showed that GOX1122 is coding for an NADP-dependent acetaldehyde dehydrogenase and they speculate that *G. oxydans* may have some kind of carbon-recovering system. The proposed reaction sequence from two molecules of acetaldehyde via acetoin to one pyruvate and one acetaldehyde would perfectly fit the purpose of acetaldehyde detoxification as well as carbon recovery in the form of CO_2_ and also matches phenotype characteristics and observed labeling data.

The labeling experiments showed an easy and fast evaluation method to estimate the participation of the two central metabolic pathways for intracellular glucose metabolism, the EDP and the PPP in *G. oxydans*. Measurement of final OD_600_ and pH after cultivation of *G. oxydans* in microtiter plates revealed small standard deviations of maximal 11%. Thus, growth of *G. oxydans* and the tested deletion mutants seems to be highly reproducible and labeling of glucose does not have an influence on growth. Cultivation in microtiter plates can be used for *G. oxydans* as a small-scale evaluation method, as it is used for other bacteria [32].

The easy sampling and biomass processing to obtain the labeling data from proteinogenic amino acids is a prerequisite for setting up automated sample treatment. With increasing availability of whole genomes and software-supported generation of metabolic carbon transition maps, this could become a valuable method for a fast and detailed screening to discriminate activity of central carbon metabolic or other pathways. The presented labeling data show that highly valuable flux estimations were obtained in a fast and reliable manner. In addition, new insight into the metabolism around the pyruvate node in *G. oxydans* was obtained, leading to a substantial hypothesis on the acetaldehyde detoxification in *G. oxydans*.

## 4. Experimental Section

### 4.1. Materials

Chemicals were obtained from Sigma-Aldrich (Taufkirchen, Germany), Qiagen (Hilden, Germany), Merck (Darmstadt, Germany), and Roche Diagnostics (Mannheim, Germany). ^13^C-labeled glucose was obtained from Cambridge Isotope Laboratories (Andover, MA, USA) with a ^13^C isotopic purity of >99% per carbon atom.

### 4.2. Bacterial Strains, Media, and Growth Conditions

The bacterial strains used in this study are listed in Table 3. *Gluconobacter oxydans* ATCC 621H Δ*upp* (ATCC 621H is identical to DSM2343), which lacks the *upp* gene for uracil phosphoribosyltransferase, was obtained from Armin Ehrenreich, Technical University of Munich, Germany [25]. In-frame deletion of the genes *edd* (GOX0431), *eda* (GOX0430), and *gnd* (GOX1705) was performed as described recently [9]. Precultures of *G. oxydans* strains were cultivated in a complex medium containing 5 g·L^−1^ yeast extract, 2.5 g·L^−1^ MgSO_4_ × 7 H_2_0, 1 g·L^−1^ (NH_4_)_2_SO_4_, 1 g·L^−1^ KH_2_PO_4_, 220 mM (4% *w*/*v*) mannitol, and 10 mM thymidine in baffled shake flasks at 30 °C and 140 rpm for 24 h. The initial pH value of the medium was 6.0. As a precaution to prevent bacterial contamination, cefoxitinwas added to the media at a concentration of 50 μg·mL^−1^ due to the natural resistance of *G. oxydans*.

For labeling experiments, cells were cultivated in 48-well, flower-shaped microtiter plates (MTP) in a BioLector^®^ (m2p-labs, Aachen, Germany). Each well was filled with 1 mL of a defined medium consisting of 1.98 g·L^−1^ (NH_4_)_2_SO_4_, 1 g·L^−1^ MgSO_4_ × 7 H_2_O, 3.48 g·L^−1^ KH_2_PO_4_, 10.5 g·L^−1^ sodium citrate, 0.5 g·L^−1^ FeSO_4_ × 7 H_2_O, 20 g·L^−1^ CaCl_2_, 50 mL·L^−1^ vitamin-Stock 100× (Sigma R 7256), 50 μg·mL^−1^ cefoxitin, and 20 mL·L^−1^ of an amino acid stock containing the 14 proteinogenic amino acids histidine, cysteine, arginine, methionine, isoleucine, phenylalanine, glutamine, proline, glycine, serine, threonine, lysine, asparagine, and glutamic acid, each at a concentration of 50 mM. The amino acids leucine, valine, and alanine were omitted to reduce interference with ^13^Clabeling signals for intracellular alanine. The initial pH was set to 6.0 using 10 M NaOH. To prevent the pH from dropping below 4, 150 mM piperazine-1,4-bispropanesulfonic acid was added as a buffer. As the primary carbon source, glucose was added at a concentration of 15 g·L^−1^ in these labeling states: naturally labeled, uniformly [^13^C]), [1-^13^C]-, and [1,2-^13^C]-labeled.

**Table 3:** Strains used in this study.

Strain (*G. oxydans*)	Description	Source
Δ*upp* Δ*gnd*	Δ*gnd* derivative of *G. oxydans* 621H Δ*upp*	[9]
	(Deletion of GOX1705)	
Δ*upp* Δgnd-zwf*	Δgnd-zwf* derivative of *G. oxydans* 621H Δ*upp*	[9]
	(Deletion of GOX1705) with mutation in *zwf* (GOX0145)	
Δ*upp* Δ*edd-eda*	Δ*edd-eda* derivative of *G. oxydans* 621H Δ*upp*	[9]
	(Deletion of GOX0430 and GOX0431)	
Δ*upp*	Δ*upp* derivative of *G. oxydans* 621H	[16]
	(Deletion of GOX0327)	

Cells of the preculture were centrifuged for 3 min at 10,414 × *g* and 4 °C. The supernatant was discarded and the remaining cell pellet was washed twice with 5 mL 0.9% NaCl. The MTPs were inoculated to an OD_600_ of 0.10 ± 0.02 and incubated at 30 °C for 24 h. The shaking frequency was set at 1000 rpm to avoid oxygen limitation, which had been checked in preliminary experiments with online pO_2_ determination by optode measurement. Evaporation is minimized by relative humidity of 100% in the incubation chamber and sealing of MTPs with gas-permeable membranes (m2p-labs). Each combination for substrate and mutant strain was cultivated in at least two biological and three technical replicates, whereof two were used for biomass hydrolysis and one for offline measurement of process data.

### 4.3. Sample Treatment and Analysis

The cells were harvested after 24 h from MTP and centrifuged for 10 min with 10,414 *× g* and the supernatant was stored for HPLC analysis or glucose assay. The unwashed cell pellet was suspended in 200 μL of 6 M HCL and incubated at 110 °C for 24 h in airtight sealed reaction cups. After cooling down to room temperature, 1 mL of distilled H_2_O was added and the sample was filtered with 0.2 μm cellulose acetate membranes to remove cell debris. The clarified sample solution contained a mixture of proteinogenic amino acids and was used for LC-MS/MS analysis of L-alanine labeling pattern.

Quantification of acetate was performed by HPLC using an Agilent 1100 system (Agilent, Waldbronn, Germany) with cation exchange chromatography (Organic Acid Resin 300 × 8, CS-Chromatographie Service, Langerwehe, Germany) and diode array detection at 215 nm. As solvent, 0.005 M H_2_SO_4_ was used at a flow rate of 0.5 mL·min^−1^. The temperature was set to 40 °C and all samples were appropriately diluted to be in the linear range (0.5–5 mM) of the external calibration function. Glucose concentration was measured enzymatically with a standard protocol using hexokinase/glucose-6-phosphate dehydrogenase system with photometric detection of NADPH increase as described in [10].

### 4.4. LC-MS/MS Measurements

LC-MS/MS measurements were performed with an Agilent 1100 HPLC system (Agilent, Waldbronn, Germany) connected to an API 4000 triple quadrupole mass spectrometer (Ab Sciex Instruments, Darmstadt, Germany). For chromatographic separation, a Luna SCX column (Penomenex, Aschaffenburg, Germany) was used at 60 °C with a flow of 0.4 mL·min^−1^ [15]. For each sample 10 μL was injected. An isocratic elution with a solvent at pH 6 consisting of 5% acetic acid (in water) and 15 mM ammonium acetate was used. The samples were diluted with pure MeOH by a factor of 2 prior to injection.

### 4.5. ^13^C Labeling Nomenclature

**Figure 6:**
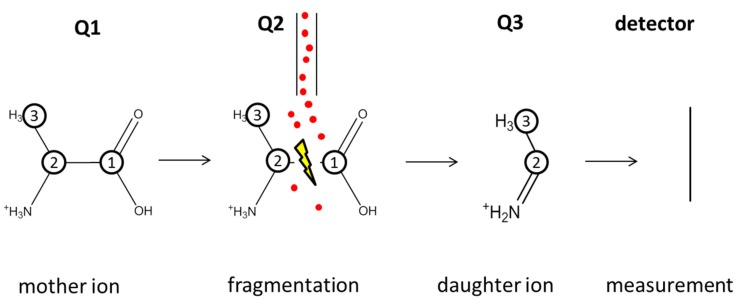
Selection and fragmentation of an ionized alanine molecule in the used triple quadrupole MS. The circled numbers indicate the carbon atoms of the alanine molecule derived from the carbon position of the substrate glucose; the red dots symbolize the inert gas particles (N_2_) for collision with the mother ion.

The index number at the carbon atom indicates its position in the carbon backbone based on IUPAC nomenclature *i.e.*, naturally labeled, uniformly [^13^C], [1-^13^C]-, and [1,2-^13^C]-labeled. The selection and fragmentation of alanine in a triple quadrupole MS are shown in Figure 6.

In the first quadrupole, the so-called mother ions of alanine are selected, followed by fragmentation in the second quadrupole; finally, the resulting daughter fragment is selected in the third quadrupole and subsequently detected. With such a measurement of the mass-to-charge ratio *(m/z)* of the mother and daughter ions, the amount of ^13^C-labeled carbons in an alanine molecule can be determined. To distinguish between the different states of ^13^C labeling, the mass isotopomer nomenclature m.x.y is used, where x is the amount of ^13^C-labeled atoms in the mother ion and y in the daughter ion. Due to the described fragmentation, the m.0.0 lane represents alanine containing only ^12^C carbon, whereas m.3.2 describes three ^13^C-labeled carbon atoms in an alanine molecule. For some labeling states, it is possible to gain additional information about the ^13^C labeling position in the carbon backbone from the available MS/MS fragmentation data. For example, an alanine molecule with ^13^C labeling at the specific carbon position C1 results in a m.1.0 signal, whereas ^13^C labeling at the position C2 or C3 results in an m.1.1 signal. To make the results shown in Table 1 more tangible, the labeling states were lumped together as unlabeled, single-, double-, and triple-labeled mass isotopomer fractions (m.0, m.1, m.2, and m.3).

### 4.6. Washout Correction

At the beginning of the ^13^C labeling phase, some residual ^12^C carbon species are still present in the cultivation, resulting from the inoculum cells. To compensate for that, a standard approach for washout correction was performed [33]. With the measured biomass concentration of each cultivation at the beginning and end of the experiments, it was possible to individually correct the labeling data based on the measured natural labeling pattern of alanine (m.0 = 95.9%, m.1 = 4.1%).

## 5. Conclusions

For the first time, a ^13^C-labeled substrate together with down-scaled microtiter plate cultivation was applied for *G. oxydans* to elucidate pathway operation, exhibiting reasonable labeling costs and allowing for sufficient replicate experiments. The *in vivo* contribution of the EDP and PPP pathway to glucose catabolism was investigated. In our approach we applied specifically ^13^C labeled glucose, whereby a labeling pattern in alanine was generated intracellularly revealing a dynamic growth phase-dependent pathway switch. Increased activity of EDP was observed in the first phase with glucose excess, while PPP operation was observed in the second growth phase metabolizing the intermediary formed gluconate. This pathway switch may be a result of cellular energy demand, since PPP provides more efficient energy regeneration. Furthermore, positional ^13^C labeling of alanine indicates an unusual carbon transition, fueling the hypothesis that there is an unexpected cyclic reaction sequence around the pyruvate node in *G. oxydans*. Evidence for a growth phase-independent decarboxylation-carboxylation cycle around the pyruvate node was obtained from ^13^C fragmentation patterns of alanine. The proposed reaction sequence converts acetaldehyde into pyruvate and might have function to decrease toxic side effects of acetaldehyde in the metabolism of *G. oxydans*.
